# Response to comments on “Effectiveness of transitioning from omalizumab to dupilumab in chronic spontaneous urticaria patients with inadequate response to omalizumab”^☆^

**DOI:** 10.1016/j.waojou.2025.101142

**Published:** 2025-11-19

**Authors:** Koremasa Hayama, Mana Ito-Watanabe, Hideki Fujita

**Affiliations:** Division of Cutaneous Science, Department of Dermatology, Nihon University School of Medicine, and Center for Allergy, Nihon University Itabashi Hospital, Tokyo, Japan

To the Editor:

We sincerely appreciate the thoughtful comments regarding our recent study on the effectiveness of dupilumab in patients with chronic spontaneous urticaria (CSU) who had an inadequate response to omalizumab.^1^ We fully agree that, given the small sample size, it is premature to draw definitive conclusions about the effectiveness of dupilumab in this population. Nevertheless, we would like to emphasize several points that reflect the real-world nature of our data and clinical practice in Japan.

First, as this was a real-world observational study, omalizumab and antihistamines were not used in strict accordance with standard protocols. In Japan, the use of antihistamines is approved only up to twice the amounts of standard doses, which limits treatment escalation compared with other regions where up to fourfold dosing is permitted.^2^ Similarly, extension of omalizumab dosing intervals is often influenced by patient preference and the high cost. In addition, patients receiving the drug every 4 weeks may sometimes receive 2 injections within a five-week month, leading to a mismatch between the number of doses administered and the number of months of treatment. In our experiences, many patients who had achieved good responses after approximately 6 injections of omalizumab requested extension of dosing intervals or discontinuation, which may have affected our patient profiles. We also recognize that these points are limitations of our study.

Second, regarding the duration of dupilumab treatment, we acknowledge that pivotal trials assessed efficacy up to 24 weeks, while the duration of our observation was 16 weeks.^3^ Although a longer observation period would have been ideal, because of the high cost of dupilumab, many patients were unwilling to continue beyond this timeframe. Thus, our findings reflect not only drug effectiveness but also the realities of treatment acceptance in practice.

Third, while the figure shows overlapping effects of omalizumab and dupilumab, it should be noted that omalizumab has a half-life of approximately 26 days,^4^ and complete clearance typically requires about 5 half-lives (around 130 days), a commonly used pharmacokinetic washout period criterion in clinical trials. Therefore, after 4 months of dupilumab treatment, omalizumab is no longer expected to remain clinically effective in the patients. We thus consider the observed improvements during this period as the effect of dupilumab alone, although we cannot rule out the possibility that a transient additive effect occurred during the early phase of dupilumab therapy.

Finally, with respect to outcome measures, it is correct that the minimal clinically important difference (MCID) for the Urticaria Control Test (UCT) is 3 points.^5^ However, the UCT primarily reflects the degree of disease control, and current international guidelines define well-controlled CSU as UCT ≥12, which represents the first milestone at which disease control can be considered satisfactory.^6^ Therefore, in real-world clinical practices, achieving a UCT score of 12 or higher should be considered the optimal therapeutic target, as this threshold better reflects meaningful clinical improvement and enhanced quality of life of the patients.

In conclusion, although our study has limitations in sample size and observational design, it provides valuable insights into the real-world use of biologics in CSU. We hope that our findings will contribute to ongoing discussions and encourage further prospective studies with larger cohorts to better define the usefulness of dupilumab in patients with CSU who do not respond adequately to omalizumab.

## Use of Generative AI or AI technologies in the development of the manuscript

Nothing to declare.

## Conflict of interest declaration

Hayama K has received study grants or honoraria for speaking engagements from Boehringer-Ingelheim, Kaken Pharmaceutical, Kyorin, Kyowa-Kirin, Maruho, Mitubishi-Tanabe, Novartis, Sanofi, and Taiho.

Ito-Watanabe M has no conflicts of interest.

Fujita H has received study grants or honoraria for speaking engagements from Boehringer-Ingelheim, Kaken Pharmaceutical, Kyorin, Kyowa-Kirin, Maruho, Mitubishi-Tanabe, Novartis, Sanofi, and Taiho.
